# Isopropyl alcohol inhalation versus 5-HT_3_ antagonists for treatment of nausea: a meta-analysis of randomised controlled trials

**DOI:** 10.1007/s00228-023-03560-x

**Published:** 2023-09-14

**Authors:** James S. Kimber, Joshua G. Kovoor, John M. Glynatsis, Samuel J. West, Thi Thien Nhi Mai, Jonathan Henry W. Jacobsen, Christopher D. Ovenden, Stephen Bacchi, Joseph N. Hewitt, Aashray K. Gupta, Suzanne Edwards, Fiona J. Taverner, David I. Watson

**Affiliations:** 1 Faculty of Health and Medical Sciences, University of Adelaide, Adelaide, SA 5000 Australia; 2 Research, Audit and Academic Surgery, Royal Australasian College of Surgeons, Adelaide, SA Australia; 3 Central Adelaide Local Health Network, Adelaide, SA Australia; 4 Adelaide Health Technology Assessment, School of Public Health, University of Adelaide, Adelaide, SA Australia; 5 Department of Anaesthesia, Flinders Medical Centre, Adelaide, SA Australia; 6 College of Medicine and Public Health, Flinders University, Adelaide, SA Australia; 7 Department of Surgery, Flinders Medical Centre, Adelaide, SA Australia

**Keywords:** Aromatherapy, Isopropyl alcohol, Nausea, 5-HT3 antagonist

## Abstract

**Purpose:**

Nausea is a common and unpleasant sensation for which current therapies such as serotonin (5-HT_3_) antagonists are often ineffective, while also conferring a risk of potential adverse events. Isopropyl alcohol (IPA) has been proposed as a treatment for nausea. We aimed to compare IPA with 5-HT_3_ antagonists for the treatment of nausea across all clinical settings.

**Methods:**

MEDLINE, EMBASE, PubMed, CENTRAL and CINAHL were searched from inception to 17 July 2023 for randomised controlled trials (RCTs) comparing inhaled IPA and a 5-HT_3_ antagonist for treatment of nausea. Severity and duration of nausea, rescue antiemetic use, adverse events and patient satisfaction were the outcomes sought. Risk of bias (RoB) was assessed using Cochrane RoB 2. Random-effects model was used for meta-analysis. Combination of meta-analyses and narrative review was used to synthesise findings. The evidence was appraised using GRADE.

**Results:**

From 1242 records, 4 RCTs were included with 382 participants. Participants receiving IPA had a significantly lower mean time to 50% reduction in nausea (MD − 20.06; 95% CI − 26.26, − 13.85). Nausea score reduction at 30 min was significantly greater in the IPA group (MD 21.47; 95% CI 15.47, 27.47). IPA led to significantly reduced requirement for rescue antiemetics (OR 0.60; 95% CI 0.37, 0.95; *p* = 0.03). IPA led to no significant difference in patient satisfaction when compared with a 5-HT_3_ antagonist. The overall GRADE assessment of evidence quality ranged from very low to low.

**Conclusion:**

IPA may provide rapid, effective relief of nausea when compared with 5-HT_3_ antagonists.

**Supplementary Information:**

The online version contains supplementary material available at 10.1007/s00228-023-03560-x.

## Background

Nausea is a feeling of sickness associated with the urge to vomit [1] and has a significant impact on the patient experience and healthcare worldwide [2, 3]. Nausea and its sequelae can range from mildly uncomfortable to severe [4–6]. There are severe clinical difficulties in treating nausea, as measures of severity are limited to subjective patient-reported scales [7]. Current treatment of nausea usually comprises antiemetic medication, most commonly antagonists of histamine (H_1_), dopamine (D_2_) or serotonin (5-HT_3_) receptors [8]. 5-HT_3_ antagonists are frequently employed as a first-line treatment [9]. Although these treatments are widely used and considered to be the most effective agents in the post-surgical setting, they are sometimes ineffective, can be associated with adverse events and can incur sizeable financial costs for healthcare [10–15]. Deleterious side effects associated with drugs of the 5-HT_3_ antagonist class include headaches, constipation, abdominal pain and QTc prolongation [16, 17].

The interactions and pathways that cause nausea and vomiting are complex. Nausea is the conscious awareness of the subconscious stimulation of the part of the medulla adjacent to or part of the emetic centre. This can be caused by impulses from as follows: the chemoreceptor trigger zone (CTZ) in the nucleus tractus solitarius of the area postrema located in the midbrain, the cerebral cortex or, directly, the gastrointestinal tract [18].

Aromatherapy is the inhalation of vaporised substances to alleviate symptoms. Various aromatherapies have been recommended for the treatment of nausea [19, 20]. Isopropyl alcohol (IPA), the active component of cleaning alcohol wipes commonly found in clinical settings, is one such substance [21–23]. It is non-invasive, inexpensive, associated with minimal adverse events [24] and readily available across healthcare settings. The exact mechanism of action of IPA aromatherapy is not well understood. It may potentially be due to its depressant effects on the central nervous system [25]. It has been suggested that inhaled IPA may regulate neuropathways involved in the emetic reflex [26]. Peak anti-nausea effect appears to be reached at around 4 min post-inhalation [19] and continued short-lasting relief may be provided by repeated inhalation of vapour [27].

The effectiveness and safety of IPA for the treatment of nausea is not widely understood, and comparison with 5-HT_3_ antagonists has been limited. Although the effectiveness of aromatherapy on post-operative nausea and vomiting has been the subject of previous review [28], the use of IPA has not previously been compared with 5-HT3 antagonists across clinical settings. To determine the potential for IPA to be used for nausea in the clinical setting, we performed a systematic review and meta-analysis of randomised controlled trials (RCTs) comparing IPA inhalation to 5-HT_3_ antagonists for the treatment of nausea.

## Methods

The methods for this study—including the review question, search strategy, inclusion and exclusion criteria and risk of bias assessment—were outlined within a protocol developed prior to the conduct of the review. This protocol was prospectively registered with PROSPERO (number CRD42021259620) and followed the Preferred Reporting Items for Systematic Reviews and Meta-Analyses 2020 (PRISMA 2020) [29] reporting guidelines. Cochrane methods [30] were utilised along with a GRADE approach (proposed by the Grading of Recommendations, Assessment, Development and Evaluation (GRADE) Working Group) [31].

### Search strategy and selection criteria

The PICO (population, intervention, comparator group, outcome) framework was used to formulate the research question and inclusion criteria. This review included prospective RCTs that measured the effect of both inhaled isopropyl alcohol and a 5-HT_3_ antagonist for the treatment of nausea, vomiting or both. Studies were excluded that compared treatments for the prophylaxis of nausea. The population comprised patients of all age groups (adult and paediatric) with nausea, in any clinical setting. The intervention was IPA inhalation via any technique. No limits were placed on number of breaths, number of repetitions or time intervals between inhalations. The comparator was 5-HT_3_ antagonists. The outcomes were severity of nausea or vomiting and rates of adverse effects.

A search was performed in PubMed (incorporating MEDLINE), Embase, CINAHL and the Cochrane Library (CENTRAL) databases from inception to 17 July 2023. No language, date or publication limits were set. Searches were supplemented by consultation of current contents, reviews and original research relating to the treatment of nausea identified through targeted searches of Google Scholar and PubMed. These are detailed in Doc 1: Search Strategies.

### Data extraction

Screening of titles and abstracts was facilitated through use of a web application (Rayyan, Qatar Computing Research Institute, Ar-Rayyan, Qatar) [32]. Two reviewers independently screened titles and abstracts and then reviewed full texts. Disagreements were resolved by consensus. Three independent reviewers extracted relevant data from the included studies. These included research design, study setting, population characteristics, intervention characteristics, comparator characteristics, timeframe for follow-up, quantitative and qualitative outcomes, source of funding, reported conflicts of interest and methodological quality information. Data were synthesised in narrative and tabular formats. The primary outcomes were severity and duration of nausea, vomiting or both, after treatment as measured by a numerical scale. Other outcomes of interest included adverse effects, requirement of additional pharmacological anti-emetic intervention (rescue therapy) and patient satisfaction as measured with a validated scale.

### Data analysis

Data analyses were performed using Stata Statistical Software: Release 15.1 College Station, TX: StataCorp LP. In this meta-analysis, for the continuous outcomes, mean differences with 95% confidence intervals (CIs) were calculated, and for the dichotomous outcome: needing nausea rescue; odds ratios (ORs) and 95% CIs were calculated for each study, and then for all the studies combined. An outcome was meta-analysed if at least two of the studies reported the outcome. The *I*^2^ statistic was used to evaluate heterogeneity (with I^2^ > 50% indicating significant heterogeneity) as was Cochran’s Q *p* value (with *p* value < 0.05 indicating significant heterogeneity). A random-effects model was used for all meta-analyses. A statistical significance level of 5% was adopted. Funnel plots or Egger’s tests were not presented due to lack of power with less than 10 studies. Intended subgroup analyses comparing adult and child populations were unable to be performed owing to limited data.

Risk of bias assessments were independently performed by two reviewers. The included trials were critically appraised using the Cochrane risk-of-bias tool for randomized trials (RoB 2) [30, 33]. The authors assessed risk of bias under the following domains: randomisation process, deviations from intended interventions, missing outcome data, measurement of the outcome and selection of the reported result. The certainty of evidence was assessed using the GRADE approach. GRADEpro GDT software was used to rate evidence and present it within a GRADE evidence profile, consisting of a certainty assessment and summary of findings table [34, 35].

### Patient and public involvement

Patients and the public were not involved in the design, conduct, reporting or dissemination plans of our research.

## Results

**Fig. 1:**
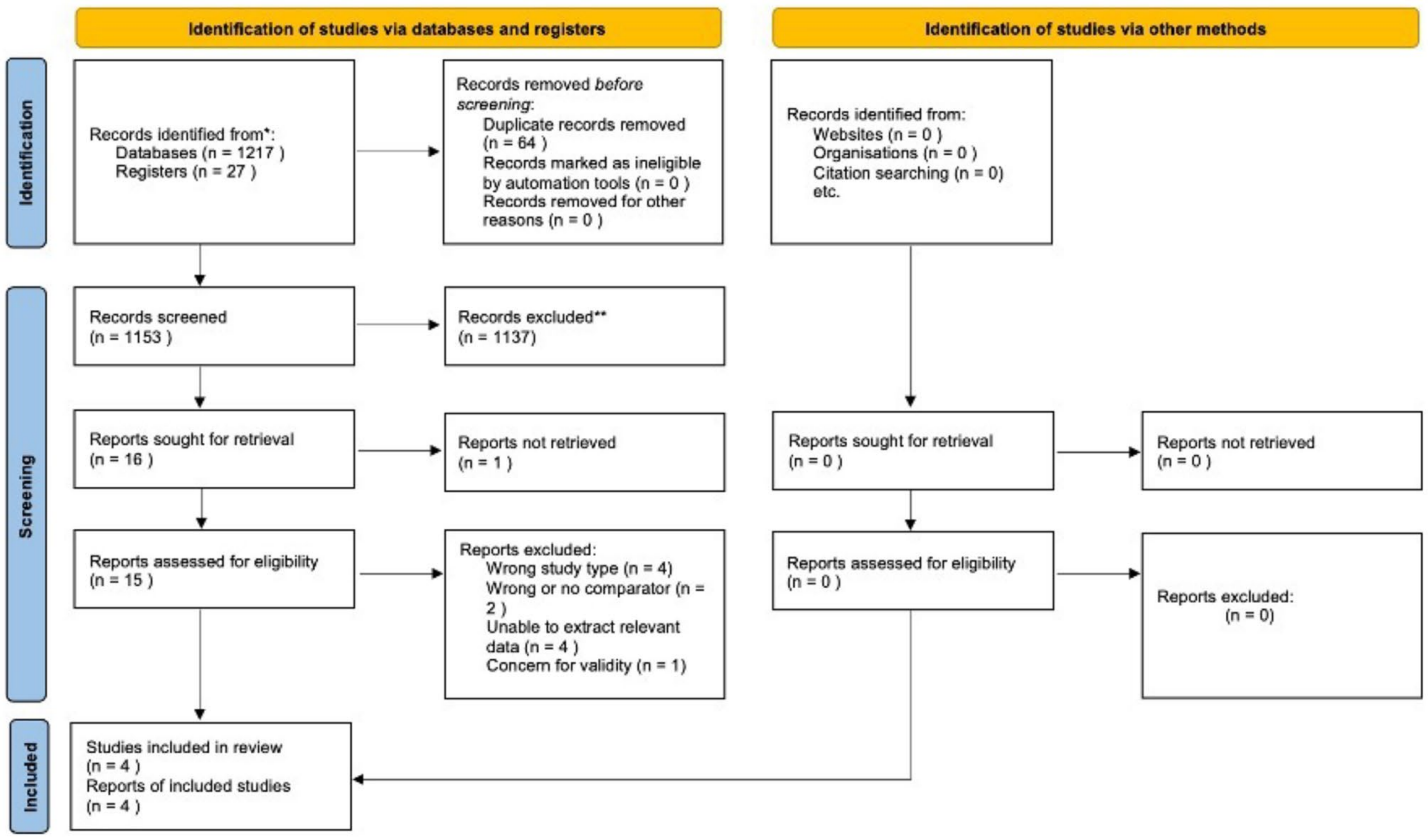
Study selection

Our searches identified 1242 records, reduced to a total of 1178 after removal of duplicates. Of these, 16 were deemed relevant to the selection criteria after title and abstract screening. The subsequent full-text appraisal identified 4 studies that met the inclusion criteria. The study selection process is summarised in Fig. 1. Of the 16 retrieved full-text studies, 10 were excluded for failing to meet the inclusion criteria; either by study type, incorrect comparator, for lacking the relevant data for meta-analysis or for inability of the reviewers to find the full text. One full text could not be obtained [36]. One study was excluded due to concern for validity of study results [37]. Further details are displayed in the Characteristics of excluded studies table (Table 1) [25, 36–46]. Of the included four studies, each was a RCT [27, 47–49], and a total of 382 study participants were included in the final study cohort. Not all trials described patient demographics. Only one study reported a source of funding. Regarding the clinical setting, two of the studies were undertaken in the postoperative setting, and two in Emergency Departments (ED). The two postoperative studies included female patients who were not critically unwell (American Society of Anesthesiology class I, II or III [47, 48]), and undergoing elective laparoscopic gynaecological surgery. The postoperative studies each controlled for anaesthesia regimen. One of the included studies examined a younger cohort (maximum age limit at 65 years of age [48]) while the others included all adults. Further details can be found in the Characteristics of included studies table (Table 2).

**Table 1:** Characteristics of excluded studies

**Study**	**Reason for exclusion**
**Dalrymple** [38]	Wrong study type — abstract
**Lindblad et al.** [39]	Wrong study type — not RCT
**Merritt et al. ** [25]	Unable to extract data relevant to review outcomes
**Radford et al.** [40]	Unable to extract data relevant to review outcomes
**Steurer ** [36]	Full text not found
**Veldhuis et al.** [41]	Wrong comparator
**Verma et al. ** [42]	Wrong comparator
**Allam ** [37]*	High level of concern for validity
**Teran and Hawkins** [43]	Unable to extract data relevant to review outcomes
**Shilpa et al.** [44]	Unable to extract data relevant to review outcomes
**Van Vooren and Semahge** [45]	Wrong study type — review
**Inhaled isopropyl alcohol for treatment of nausea**	Wrong study type

Firstly, it is published in a Longdom/OMICS journal, a recognised predatory publisher in which many papers are fabricated or unreliable^a^. Secondly, there is only one author on a paper involving considerable data collection from many subjects which is extremely unlikely. Thirdly, amongst the various tables almost every row has at least two numbers that differ only by 1, many rows being perfect arithmetic progressions. Five other papers by this author have been identified as fraudulent on PubPeer^b–f^

^a^Masic I (2017) Predatory Publishing—Experience with OMICS International. Med Arch 71(5):304–307 https://doi.org/10.5455/medarh.2017.71.304-307

^b^PubPeer (2022) Comparative study between action vitamin C vs. action of nitric oxide in prolonged ventilation in respiratory failure patients due to ARDS. In: ed

^c^PubPeer (2022) Extra corporal membrane oxygenation (ECMO) vs. conventional ventilation with nitric oxide in ARDS due to infected contused lung. In: ed

^d^PubPeer (2022) The effect of use of anidulafungin on failure of weaning due to ventilator-associated pneumonia which copmlicated contused lungs. In: ed

^e^PubPeer (2022) Comparative study between usage of meropenem/gentamicin versus ceftazidime/avibactam in the treatment of ARDS induced by both lung trauma and VAP. In: ed

^f^PubPeer (2022) Comparative study between usage of tazocin/vancomycin versus tazocin/linezolide in treatment of traumatized contused lung. In: ed. PubPeer

*The concern for fabrication is derived from several points

**Table 2:** Characteristics of included studies

**Study; country**	**Inclusion criteria; sample size**	**Design; clinical scenario**	**Intervention; comparator**	**Relevant outcomes**
Winston et al. [47] USA	Women aged > 18 Laparoscopic same-day surgery ASA class I, II or III *n* = 41	RCT, single-centre Treatment of PONV	Inhaled IPA Ondansetron (4 mg IV)	Nausea scores (VNRS) Time to 50% reduction of nausea Rescue antiemetic requirement
Cotton et al. [48] USA	Women aged 18–65 Laparoscopic same-day surgery ASA I, II or III *n* = 49	RCT, single-centre Treatment of PONV	Inhaled IPA Ondansetron (4 mg IV)	Nausea scores (VNRS) Time to 50% reduction in nausea Rescue antiemetic requirement Patient satisfaction
Kakhki et al. [49] Iran	Patients referred to ED due to isolated head trauma with nausea Excluded those with evidence of moderate-severe injury	RCT, single-centre Treatment of nausea in ED	Inhaled IPA Ondansetron	Nausea scores (VNRS) and vomiting
April et al. [27] USA	Age ≥ 18 Presenting to ED with chief complaint related to nausea or vomiting Nausea severity ≥ 3/10 (VNRS) *n* = 82	RCT, single-centre Treatment of nausea in ED	Inhaled IPA Ondansetron (4 mg ODT)	Nausea scores (VAS) Rescue antiemetic requirement Patient satisfaction

*ASA* American Society of Anesthesiologists class, *IV* intravenous, *ODT* orally disintegrating tablet, *ED* emergency department

Measures of treatment effect were mostly limited to patient-reported outcomes which were measured by scales including Verbal Numerical Rating Scales (VNRS) and Visual Analog Scales (VAS) [7, 50, 51]. Also considered was the use of additional pharmacological ‘rescue’ antiemetics. The studies used a variety of comparisons. Diversity of time points and measurement scales limited the data that could be pooled. No studies were excluded from risk of bias assessments.

### Nausea duration

**Fig. 2:**
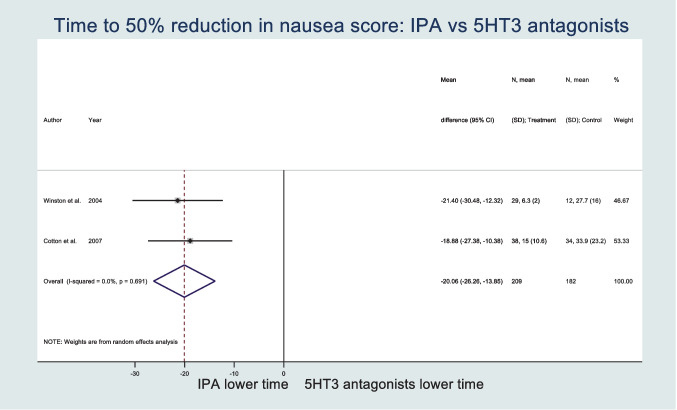
Forest plot of time to 50% reduction in nausea score

Two studies with 90 participants compared time to 50% nausea reduction between treatment with inhaled IPA and a 5-HT_3_ antagonist. The overall mean difference in time to 50% reduction in nausea score across the studies was − 20.06 min (95% CI − 26.26, − 13.85) (see Fig. 2). The overall mean time to 50% reduction in nausea score in the IPA group was 20.06 min less than that in the 5-HT_3_ antagonist group. Heterogeneity in the study estimates assessed using the I-squared statistic was 0% and Cochran’s Q *p* value was 0.691, suggesting no significant heterogeneity.

### Nausea severity

**Fig. 3:**
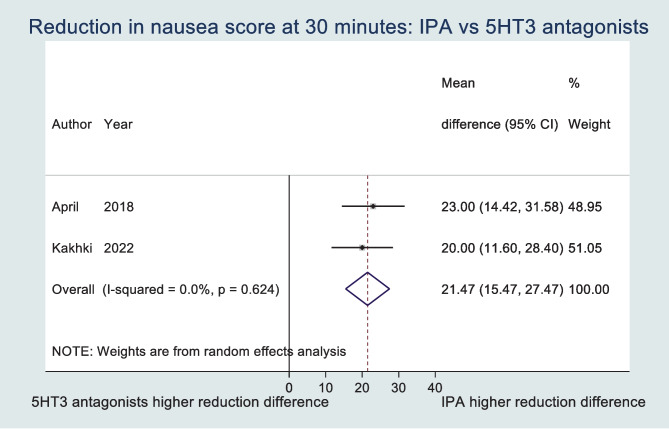
Forest plot of reduction in nausea score at 30 minutes

The severity of nausea, as recorded on a validated scale, was reported in three of the studies. Two of these studies reported a ‘reduction in nausea’ score at 30 min following treatment. The pooled mean difference in nausea reduction at 30 min was 21.47 (95% CI 15.47, 27.47) (see Fig. 3). I-squared statistic of 0% and Cochran’s Q *p* value of 0.62 indicated absence of heterogeneity. April et al. [27] also found that IPA led to a 13 points lower mean nausea score (VAS) at disposition (95% CI 3, 23). Kakhki et al. [49] also examined nausea scores at 10 min. and the presence of vomiting at 10 and 30 min, and found no significant difference between groups with respect to vomiting or nausea at 10 min, but significantly more frequent vomiting in the IPA group at 30 min (38% vs 6%, *p* = 0.001). Winston et al. [47] reported nausea scores at specific time points post-treatment (5, 10, 15, 30, 45 and 60 min). These authors found that inhaled IPA led to significantly lower median VNRS scores of nausea at the 5-, 10- and 15-min marks when compared with a 5-HT_3_ antagonist (*p* values = 0.002, 0.015 and 0.036 respectively).

### Rescue antiemetic requirement

**Fig. 4:**
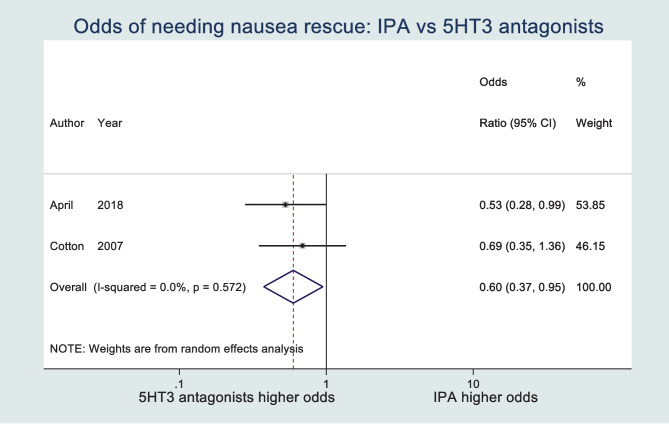
Forest plot of odds of needing nausea rescue

The use of additional ‘rescue’ antiemetic therapy was compared in two of the included studies. The odds ratio of needing nausea rescue across the studies is 0.60 (95% CI 0.37, 0.95; *p* = 0.03) in favour of IPA, reaching the nominal statistical significance threshold. *I*-squared statistic was 0%, and Cochran’s Q *p* value was 0.57 which show absence of heterogeneity (see Fig. 4).

### Adverse events

One study reported on the effect of nausea treatment with inhaled IPA or a 5-HT_3_ antagonist on adverse events, reporting no recorded adverse events in either group [27].

### Patient satisfaction

The effect of nausea treatment method on patient satisfaction was reported by two included studies. April et al. [27] reported on overall patient satisfaction as a mean score (VAS), finding no significant difference between those exposed to IPA rather than 5-HT_3_ antagonist. Cotton et al. [48] measured patient satisfaction on a four-point scale and found no significant difference between the groups.

### Risk of bias in studies

Assessment of risk of bias of each of the included studies was performed using the Cochrane risk-of-bias tool for randomised trials (RoB 2). The risk of bias varied across the studies, ranging from ‘some concerns’ to ‘high risk’. The RoB 2 judgements for all study results in all domains are available in the risk of bias table (Doc 3). Briefly summarised, the majority of RCTs (3/4) were considered an overall high risk of bias. The one remaining study was rated overall as ‘some concerns’ for risk of bias. A persistent area of concern was that the use of an inhaling agent precluded blinding as patients could identify which treatment they were allocated to. The Doc 2: GRADE Evidence Profile is provided in the appendix.

## Discussion

This systematic review and meta-analysis of RCTs suggests that inhaled IPA may have comparable or superior effectiveness across clinical settings for the treatment of nausea when compared with 5-HT_3_ antagonists. Nausea treated with inhaled IPA resolved significantly faster, and nausea severity reduced when compared with those treated with a 5-HT_3_ antagonist. Specifically, our meta-analysis found the mean time to 50% reduction in nausea score was 20.06 min less, and the reduction in nausea score at 30 min 21.47 points greater in patients treated with inhaled IPA when compared with a 5-HT_3_ antagonist. Rescue antiemetic requirement was significantly lower in those treated with inhaled IPA. No significant difference was found in patient satisfaction between groups. Prior to our review, none had previously compared the use of isopropyl alcohol and 5-HT_3_ antagonists in the treatment of nausea in all clinical settings. However, our evidence base largely reflects the use of antiemetics in the postoperative and emergency department settings.

The findings of improved nausea duration associated with inhaled IPA when compared with 5-HT_3_ antagonists support the suggested utility of using this treatment method [26]. A systematic review of the effectiveness of aromatherapies for postoperative nausea and vomiting [28] found similarly that inhaled IPA appeared effective in reducing nausea duration and rescue antiemetic use when compared to ‘standard treatment’. Another systematic review comparing the effectiveness of complementary non-invasive therapies on postoperative nausea and vomiting in women undergoing laparoscopic hysterectomy [52] found that inhaled IPA when compared with a 5-HT_3_ antagonist was effective in altering duration of nausea; however, it did not significantly reduce the need for rescue antiemetics. The results of these reviews are mostly in agreement with our meta-analysis, in finding either equivalence between the comparators or benefit in the direction of inhaled IPA treatment.

IPA is delivered through pads soaked in the substance being placed close to the nares, through which patients inhale taking two to three deep breaths. IPA has been proven to be safe in animal models [53, 54], and no adult studies have recorded any related adverse events [22, 48]. It exhibits toxicity to human adults only with oral ingestion. Of the investigated aromatherapies, IPA has shown particularly promising results in systematic reviews [20, 52] and trials [55–57]. However, one trial found it to be equivalent to peppermint aromatherapy and placebo [58]. This suggests further placebo-controlled trials are required. IPA appears to be used for the treatment of nausea in various clinical settings, including EDs and post-anaesthesia care units [25, 41, 48, 59]. Delivery via inhalation is an advantage, particularly in post-operative patients who may be unable to swallow oral medications and be at a higher risk of aspiration if that occurs. Its use as a traditional treatment for nausea is said to have originated in South America [21, 22, 58].

There are limitations to this study. There was one text identified through title and abstract screening which could not be retrieved [36], and given the small evidence base, the absence of that trial may impact results. The meta-analyses had low levels of heterogeneity, so the risk of bias from this source was also low. The studies that showed changes in nausea severity in favour of the IPA group were generally at relatively brief time intervals. There was limited evidence for preferential effect of IPA at 60 min or greater.

In general, the studies included more patients who were female, relatively young and not critically unwell. These demographic factors may limit the generalisability of the results and add another source of bias. A potential source of bias arises from the fact that the majority of included studies in this review were published in the USA, thereby not accounting for potential regional differences in practices regarding care for nausea patients.

It is possible that further studies of inhaled IPA for the treatment of nausea with greater rigour in study methods could find differing results from those identified in this review. However, there was minimal inconsistency in the pooled results, improving our level of confidence in these findings. The evidence for inhaled IPA when compared with 5-HT_3_ antagonists in the treatment of nausea is incomplete, with no paediatric participants (age < 18) included in the review. The GRADE certainty ratings for the outcome results ranged from very low to low, with generally small sample sizes, incomplete reporting and concerning risk of bias leading to downgrading of evidence quality ratings. Inhaled IPA is inherently a difficult intervention to blind to both participants and experimenters, owing to its strong odour. Minimal inconsistency in the pooled results increases the level of confidence in the found effects. Low participant numbers in some included studies resulted in imprecision, reducing quality of evidence. However, there was little concern over publication bias. The capacity to detect bias in meta-analyses on a limited number of trials is limited [60], however, and there exists a plausibility of publication bias amongst some of these included trials which revealed perhaps surprising results. The exact method of IPA inhalation has not been yet standardised, in terms of inhaled breaths taken and intervals between inhalations, limiting comparability of the results.

Although it was not the primary focus of this analysis, the cost differences between IPA and 5-HT_3_ antagonists should be considered when evaluating their respective roles. Previous commentaries in Western settings have highlighted that IPA inhalation is inexpensive compared with 5-HT_3_ antagonists [39]. For example, ondansetron, a commonly utilised 5-HT_3_ antagonist costs $7.39 for four tablets [61]. While there may be variation between providers and methods of IPA delivery, this treatment method would typically cost less than $0.05 [41] for a routine hospital disinfectant swab. While this cost difference may seem small at an individual patient level, at a systems level, the cost difference may be significant. This difference may have particular significance for low-income settings.

When considering the implications of the results of this analysis, the utility of a multimodal approach to the management of nausea should be considered. Previous studies have identified that utilising a combination of drug therapies, along with non-pharmacological interventions, may be most effective in the management of PONV [62]. While this review focussed on comparative studies, the possible role of IPA in a multimodal approach that utilises interventions with a diverse array of mechanisms of actions may be an area that would benefit from further study.

As nausea is a frequent and significant issue in most clinical situations, especially in the postoperative setting, findings from this meta-analysis of RCTs provide valuable information for healthcare systems and providers. Comparative or superior treatment of nausea may be obtained through the administration of inhaled IPA relative to 5-HT_3_ antagonists — the current first-line treatment of nausea in many settings globally. Notably, IPA was not associated with a significant difference in patient satisfaction. Our meta-analysis suggests that IPA inhalation could be considered as a novel, effective treatment for nausea across clinical settings. However, the overall quality of the evidence included was low, and there are significant limitations to the interpretation of the findings. Future research could strengthen the literature via RCTs in paediatric populations and with larger sample sizes across multiple centres, with further data on both short- and medium-term outcomes as well as adverse effects.

### Supplementary Information

Below is the link to the electronic supplementary material.

Supplementary file1 (DOCX 245 KB)

## Data Availability

The datasets used and/or analysed during the current study are available from the corresponding author on reasonable request.

## List of abbreviations

CI: Confidence interval
CTZ: Chemoreceptor trigger zone
D2: Dopamine
ED: Emergency department
IPA: Isopropyl alcohol
GRADE: Grading of Recommendations, Assessment, Development and Evaluations
GDT: Guideline development tool
H1: Histamine
MOOSE: Meta-analysis of Observational Studies in Epidemiology
PICO: Population, intervention, comparator group, outcome
PONV: Postoperative nausea and vomiting
PRISMA: Preferred Reporting Items for Systematic Reviews and Meta-analyses
QTc: Corrected QT-interval
RCT: Randomised controlled trial
RoB: Risk of bias
VAS: Visual analogue scale
VNRS: Verbal numerical rating scale
5-HT3: Serotonin
