# Supplementary material for: Isopropyl alcohol inhalation versus 5-HT3 antagonists for treatment of nausea: a meta-analysis of randomised controlled trials
Source: Eur J Clin Pharmacol. 2023 Sep 14;79(11):1525–35. doi: 10.1007/s00228-023-03560-x (PMC10618376; doi:10.1007/s00228-023-03560-x)

**LIST OF SUPPORTING INFORMATION**

| Doc 1 | Search strategies |
| --- | --- |
| Doc 2 | GRADE Evidence Profile |
| Doc 3 | Risk of Bias Tables |

**Doc 1: Search Strategies**

**Updated searches: 10 June 2021 – 17 July 2023**

**PubMed (incorporating MEDLINE)**

("5-HT3" OR "5 HT3" OR "5HT3" OR setron OR ondansetron OR tropisetron OR granisetron OR dolasetron OR palonosetron OR ramosetron) AND ("isopropyl alcohol" OR "iso-propyl alcohol" OR "propan-2-ol" OR "2-propanol" OR isopropanol OR "sec-propyl alcohol" OR "2-hydroxypropane" OR "i-PrOH" OR "dimethyl carbinol" OR "IPA")

38 results

**Embase***

(ondansetron OR tropisetron OR granisetron OR 'dolasetron mesilate' OR palonosetron OR ramosetron OR 'serotonin 3 antagonist') AND (isopropyl OR 'iso-propyl' OR '2-propanol' OR 'propan-2-ol' OR isopropanol OR '2-hydroxypropane' OR 'ipa')

8 results

**CINAHL***

("5-HT3" OR "5 HT3" OR "5HT3" OR setron OR ondansetron OR tropisetron OR granisetron OR dolasetron OR palonosetron OR ramosetron) AND ("isopropyl alcohol" OR "iso-propyl alcohol" OR "propan-2-ol" OR "2-propanol" OR isopropanol OR "sec-propyl alcohol" OR "2-hydroxypropane" OR "i-PrOH" OR "dimethyl carbinol" OR "IPA")

3 results

**Cochrane Library (CENTRAL)**

("5-HT3" OR "5 HT3" OR "5HT3" OR setron OR ondansetron OR tropisetron OR granisetron OR dolasetron OR palonosetron OR ramosetron) AND ("isopropyl alcohol" OR "iso-propyl alcohol" OR "propan-2-ol" OR "2-propanol" OR isopropanol OR "sec-propyl alcohol" OR "2-hydroxypropane" OR "i-PrOH" OR "dimethyl carbinol" OR "IPA")

5 results

***** date limit from 2021 to 2023

**Initial searches: Inception – 10 June 2021**

**PubMed (incorporating MEDLINE)**

("5-HT3" OR "5 HT3" OR "5HT3" OR setron OR ondansetron OR tropisetron OR granisetron OR dolasetron OR palonosetron OR ramosetron) AND ("isopropyl alcohol" OR "iso-propyl alcohol" OR "propan-2-ol" OR "2-propanol" OR isopropanol OR "sec-propyl alcohol" OR "2-hydroxypropane" OR "i-PrOH" OR "dimethyl carbinol" OR "IPA")

907 results

**Embase**

(ondansetron OR tropisetron OR granisetron OR 'dolasetron mesilate' OR palonosetron OR ramosetron OR 'serotonin 3 antagonist') AND (isopropyl OR 'iso-propyl' OR '2-propanol' OR 'propan-2-ol' OR isopropanol OR '2-hydroxypropane' OR 'ipa')

220 results

**CINAHL**

("5-HT3" OR "5 HT3" OR "5HT3" OR setron OR ondansetron OR tropisetron OR granisetron OR dolasetron OR palonosetron OR ramosetron) AND ("isopropyl alcohol" OR "iso-propyl alcohol" OR "propan-2-ol" OR "2-propanol" OR isopropanol OR "sec-propyl alcohol" OR "2-hydroxypropane" OR "i-PrOH" OR "dimethyl carbinol" OR "IPA")

41 results

**Cochrane Library (CENTRAL)**

("5-HT3" OR "5 HT3" OR "5HT3" OR setron OR ondansetron OR tropisetron OR granisetron OR dolasetron OR palonosetron OR ramosetron) AND ("isopropyl alcohol" OR "iso-propyl alcohol" OR "propan-2-ol" OR "2-propanol" OR isopropanol OR "sec-propyl alcohol" OR "2-hydroxypropane" OR "i-PrOH" OR "dimethyl carbinol" OR "IPA")

22 results

**Doc 2: GRADE Evidence Profile**

| **Certainty assessment** | | | | | | | **№ of patients** | | **Effect** | | **Certainty** | **Importance** |
| --- | --- | --- | --- | --- | --- | --- | --- | --- | --- | --- | --- | --- |
| **№ of studies** | **Study design** | **Risk of bias** | **Inconsistency** | **Indirectness** | **Imprecision** | **Other considerations** | **Inhaled Isopropyl Alcohol** | **5-HT3 Antagonists** | **Relative (95% CI)** | **Absolute (95% CI)** |  |  |
| **Duration of nausea (assessed with: Time to 50% reduction of nausea score)** | | | | | | | | | | | | |
| 2 | randomised trials | very serious^a,b,c^ | not serious | serious^d^ | serious^e^ | none | 58 participants | 32 participants | **MD -20.06** (-26.26 to -13.85) [Duration of nausea] | **-- per 1,000** (from -- to --) | ⨁◯◯◯ Very low | CRITICAL |
|  |  |  |  |  |  |  | - | 15.0% |  | **-- per 1,000** (from -- to --) |  |  |
| **Severity of nausea (follow-up: 30 minutes; assessed with: Reduction in nausea score; Scale from: 0 to 100)** | | | | | | | | | | | | |
| 2 | randomised trials | serious^a,b,c^ | not serious^f^ | not serious | serious^e^ | none | 146 | 146 | - | MD **21.47 higher** (15.47 higher to 27.47 higher) | ⨁⨁◯◯ Low | CRITICAL |
| **Rescue antiemetic requirement (assessed with: Percentage requiring additional antiemetics)** | | | | | | | | | | | | |
| 2 | randomised trials | very serious^a,b,c,g^ | serious^f^ | not serious | serious^h,i^ | none | 20/70 (28.6%) | 31/53 (58.5%) | **OR 0.60** (0.37 to 0.95) | **127 fewer per 1,000** (from 242 fewer to 13 fewer) | ⨁◯◯◯ Very low | IMPORTANT |
| **Patient satisfaction (assessed with: Various scales including VAS)** | | | | | | | | | | | | |
| 2 | randomised trials | serious^a,b,c,g^ | not serious | not serious | serious^e^ | none | Two studies found no significant difference between the groups | | | | ⨁⨁◯◯ Low | IMPORTANT |
| **Adverse events** | | | | | | | | | | | | |
| 1 | randomised trials | serious^a,b,g^ | very serious^j^ | not serious | serious^k^ | none | One study reported no adverse events in either the IPA (0/41) and 5-HT3 (0/41) groups. | | | | ⨁◯◯◯ Very low | CRITICAL |
| **Severity of nausea (assessed with: various)** | | | | | | | | | | | | |
| 3 | randomised trials | serious^a,b,c^ | serious^f^ | not serious | serious^e^ | none | Three studies reporting conflicting data with IPA leading to significantly reduced nausea (2/3) or significantly greater nausea (1/3). | | | | ⨁◯◯◯ Very low | CRITICAL |

**CI:** confidence interval; **MD:** mean difference; **OR:** odds ratio

#### Explanations

a. Deviations from intended interventions

b. Significant risk of bias in the domain of measurement of the outcome

c. Significant risk of bias in blinding

d. Populations of included studies not applicable to the target population

e. Low participant numbers in studies meaning they were likely underpowered to detect differences

f. Studies reported opposing effects

g. Significant risk of bias due to missing outcome data

h. Confidence interval spans no effect

i. Results vary from appreciable harm to benefit

j. Highly varied incidence of adverse events between studies

k. Likely underpowered to detect rare events

**Doc 3: Risk of Bias Tables**


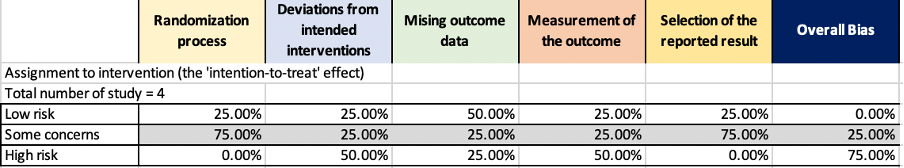


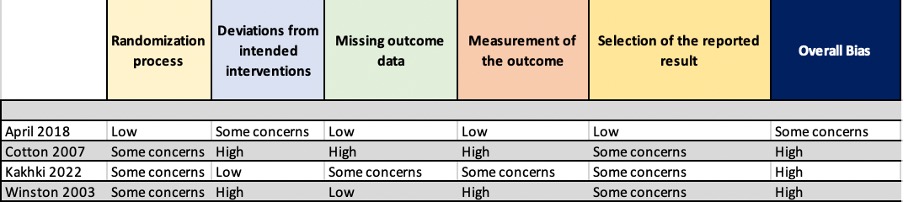

Supplement: Supplementary file 1 — Supplementary file1 (DOCX 245 KB) [file 228_2023_3560_MOESM1_ESM.docx]
